# Diagnostic Potential of Plasmatic MicroRNA Signatures in Stable and Unstable Angina

**DOI:** 10.1371/journal.pone.0080345

**Published:** 2013-11-15

**Authors:** Yuri D'Alessandra, Maria Cristina Carena, Liana Spazzafumo, Federico Martinelli, Beatrice Bassetti, Paolo Devanna, Mara Rubino, Giancarlo Marenzi, Gualtiero I. Colombo, Felice Achilli, Stefano Maggiolini, Maurizio C. Capogrossi, Giulio Pompilio

**Affiliations:** 1 Laboratory of Vascular Biology and Regenerative Medicine, Centro Cardiologico Monzino, IRCCS, Milan, Italy; 2 Department of Vascular Surgery, Centro Cardiologico Monzino, IRCCS, Milan, Italy; 3 Cardiac Intensive Care Unit - UTIC, Centro Cardiologico Monzino, IRCCS, Milan, Italy; 4 Laboratory of Immunology and Functional Genomics, Centro Cardiologico Monzino, IRCCS, Milan, Italy; 5 Statistical Center, I.N.R.C.A. National Institute, Ancona, Italy; 6 Cardiology Department, San Gerardo Hospital, Monza, Italy; 7 Division of Cardiology, S.L. Mandic Hospital, Merate, Italy; 8 Laboratory of Vascular Pathology, Istituto Dermopatico dell'Immacolata, IDI - IRCCS, Rome, Italy; 9 Department of Clinical Sciences and Community Health, Università degli Studi di Milano, Milan, Italy; National Institutes of Health, United States of America

## Abstract

**Purpose:**

We examined circulating miRNA expression profiles in plasma of patients with coronary artery disease (CAD) vs. matched controls, with the aim of identifying novel discriminating biomarkers of Stable (SA) and Unstable (UA) angina.

**Methods:**

An exploratory analysis of plasmatic expression profile of 367 miRNAs was conducted in a group of SA and UA patients and control donors, using TaqMan microRNA Arrays. Screening confirmation and expression analysis were performed by qRT-PCR: all miRNAs found dysregulated were examined in the plasma of troponin-negative UA (n=19) and SA (n=34) patients and control subjects (n=20), matched for sex, age, and cardiovascular risk factors. In addition, the expression of 14 known CAD-associated miRNAs was also investigated.

**Results:**

Out of 178 miRNAs consistently detected in plasma samples, 3 showed positive modulation by CAD when compared to controls: miR-337-5p, miR-433, and miR-485-3p. Further, miR-1, -122, -126, -133a, -133b, and miR-199a were positively modulated in both UA and SA patients, while miR-337-5p and miR-145 showed a positive modulation only in SA or UA patients, respectively. ROC curve analyses showed a good diagnostic potential (AUC ≥ 0.85) for miR-1, -126, and -483-5p in SA and for miR-1, -126, and -133a in UA patients vs. controls, respectively. No discriminating AUC values were observed comparing SA vs. UA patients. Hierarchical cluster analysis showed that the combination of miR-1, -133a, and -126 in UA and of miR-1, -126, and -485-3p in SA correctly classified patients vs. controls with an efficiency ≥ 87%. No combination of miRNAs was able to reliably discriminate patients with UA from patients with SA.

**Conclusions:**

This work showed that specific plasmatic miRNA signatures have the potential to accurately discriminate patients with angiographically documented CAD from matched controls. We failed to identify a plasmatic miRNA expression pattern capable to differentiate SA from UA patients.

## Introduction

The amount of patients hospitalized in Western countries for chest pain accounts for several millions each year. In approximately half of the cases, chest pain is of cardiac origin [1]. Among these patients, approximately 50% exhibit an underlying coronary artery disease (CAD), causing either stable (SA) or unstable (UA) angina pectoris or myocardial infarction. In the absence of myocardial necrosis leading to increased plasmatic levels of the cardiac-specific protein Troponin, there are currently no established circulating biomarkers that may support the diagnosis of SA or UA. Notably, missed diagnosis of cardiac ischemia has been demonstrated to cause an increase in early and mid-term mortality [2]. Thus, the identification of novel noninvasive biomarkers of CAD lacking myocardial necrosis is a compelling need still under investigation. 

MicroRNAs (miRNAs) are ~22 nucleotides long non-coding RNAs, known to regulate complex biological process by 'fine-tuning' the translation of specific messenger RNA targets [3]. MiRNAs are pivotal modulators of mammalian cardiovascular development and disease and can be steadily found in the systemic circulation of both animals and humans, where they show a remarkable stability probably due to internalization in vesicles and binding to circulating proteins and other molecules [4]. Since their levels may significantly change upon stress, circulating miRNAs have been proposed as diagnostic biomarkers in different pathologic conditions (e.g. cancer, cardiac diseases, liver injury, and hepatitis) [4-6]. Interestingly, recent reports have suggested the diagnostic potential of miRNAs in heart diseases, such as heart failure [7] and myocardial infarction (MI) [8]. In particular, we previously reported the presence of a distinctive “signature” of 6 circulating miRNAs in patients with ST-Elevation Myocardial Infarction (STEMI) [8]. In a more recent work, we have shown that circulating miR-499-5p may be a useful marker for early discrimination between congestive heart failure and Non ST-Elevation Myocardial Infarction (NSTEMI) in elderly patients presenting to hospital with unclear symptoms [9].

In the search for diagnostic biomarkers of chest pain of cardiac origin, circulating miRNAs have been previously investigated in blood, serum, plasma, platelets, and peripheral blood mononuclear cells (PBMC) in patients with both stable and unstable angina [10]. However, the high heterogeneity in study design, patient population, and miRNA source and detection methods is likely to be responsible for the high variability and poor overlap of the aberrant miRNA signatures identified, making extrapolation of conclusive information difficult. 

The purpose of the present study was to search for distinctive miRNA profiles in plasma of patients with angiographically-documented troponin-negative CAD in comparison to controls matched for cardiovascular risk factors. The potential diagnostic significance of circulating miRNAs has been challenged by ROC curve and hierarchical cluster analysis with the aim of identifying putative specific CAD-associated miRNA signatures.

## Materials and Methods

### Study population

#### CAD Subjects

The study was approved by the Ethical Committee of Centro Cardiologico Monzino (CCM) IRCCS, Milano, Italy. All subjects provided written informed consent at the time of enrollment. Fifty-three patients affected by angiographically documented CAD were enrolled for this study. CAD was defined as at least one major epicardial vessel with >70% stenosis, assessed by quantitative coronary angiography. Subjects included in the study were both patients with UA referred to the First Aid Department (duration of symptoms from onset to hospital admission ≤ 24 hrs) and patients affected by SA recruited from the Outpatient Department. Angina pectoris was categorized as either stable or unstable according to ACC/AHA guidelines [11,12]. In particular, patients with SA had typical chest pain on exertion associated with ST segment depression >1.0 mm on an exercise test. UA was defined as chest pain occurring at rest or minimal exertion, of new onset (within 1 month) or with a worsening pattern (in frequency, intensity, or duration), without evidence of myocardial necrosis based on the rise in cardiac serum markers such as creatine kinase isoenzyme (CK)-MB and troponin T or I.

Clinical history and medication records were also collected. Exclusion criteria were: impaired left ventricular ejection fraction ≤ 45%, congestive heart failure, chronic kidney or hepatic disease, acute myocardial infarction, and malignant disease.

#### Controls

Twenty volunteers matched for sex, age, smoking habit, hypertension, and diabetes and without history of CAD or inflammatory disorders served as control group. All volunteers provided written informed consent at the time of enrollment.

### Samples Collection

Peripheral venous blood samples were collected in EDTA-coated tubes (BD) and either processed immediately or stored at 4°C for up to 24h. Previous analysis showed that the storage method does not impact on miRNA detection [8]. Cell- and platelet-free plasma was prepared following a 2 step centrifugation protocol. After plasma separation from blood, samples were centrifuged at 1.500 × *g* for 15’ at 4°C to remove remaining cells and debris. The supernatant was centrifuged again at 14.000 × *g* for 15’ at 4°C to obtain platelet-poor plasma and, thereafter, aliquoted and stored at -80°C until use.

### RNA extraction

Total RNA was extracted from 400 µl of plasma using 1 ml of TRIzol reagent (Life Technologies), following a modified protocol for liquid samples. Briefly, following phase separation, RNA was precipitated by adding 30 μg of glycogen (Roche) and 1 ml of isopropanol to the recovered aqueous phase. After 10’ at room temperature, RNA samples were pelleted 10’ at 12.000 × *g*, washed with 1 ml of ethanol 70%, and centrifuged again 10’ at 12.000 × *g*. Supernatants were eliminated and, after a 5’ drying step, pellets were resuspended in RNAse-free water.

### miRNAs expression analysis

TaqMan Human microRNA Card A Arrays version 2.0 (Life Technologies) were used for expression screening of 367 miRNAs, conducted on 4 RNA samples randomly selected from each group of subjects (SA, UA and Controls). Reverse transcription (RT) and pre-amplification steps were performed according to the manufacturer’s protocol, using a 7900HT Fast Real-Time PCR System (Life Technologies). Screening results were expressed as Ct (cycle threshold) levels and normalized to the median Ct of each sample (ΔCt), so that all subsequent calculations were independent from RNA amount added to the PCR reaction. Relative expression was calculated using the comparative Ct method (2-[Δ][Δ]Ct). All miRNAs detectable in all 4 samples showing a Ct < 40 were considered as expressed. To minimize the number of false positives, we considered for the subsequent confirmation phase only those miRNAs whose expression in CAD patients significantly differed from the controls on average more than 2-folds. All following steps were conducted by qRT-PCR using single TaqMan microRNA assays (Life Technologies), accordingly to manufacturer’s instructions. Since no RNA quantitation was possible, the same volume of RNA solution (2 µl) was inputted in each RT assay for technical consistency.

Profiling data analysis showed 4 potential normalizers that exhibited strong expression in all samples: miR-15b, miR-16, miR-17-5p, and miR-29. Among these, using a restricted cohort of randomly selected patients and controls (n=10 for each group), two miRNAs were identified as best potential normalizers using the geNorm algorithm [13]: miR-16 and miR-17-5p. After assessing that no discrepancies were detectable by the usage of either miR-16 or miR-17-5p as single normalizer (data not shown), we selected miR-16 as internal calibrator, based on its persistent and stable expression throughout all considered samples. The confirmation step of the screening results was conducted on the same samples used to identify the best internal normalizer, and then the analysis was extended to all remaining samples (24 for SA, 9 for UA and 10 for Controls). 

### Laboratory assays

Cardiac troponin I (cTnI), creatine kinase (CK) and MB isoenzyme of creatine kinase (CK-MB) were measured using the Access Immunoassay System (Beckman Coulter). Triglycerides and total, LDL and HDL cholesterol were measured using the UniCel DxC 600 (Beckman Coulter). 

### Statistical methods

Validated miRNAs expression data were analyzed using GraphPad Prism version 5.03 for Windows (GraphPad Software, San Diego, CA, www.graphpad.com) and reported as mean ± standard error of the mean (SEM). Expression data were tested for normal distribution using D’Agostino and Pearson normality test and differences among groups were compared using either One-way ANOVA or non-parametric Kruskal-Wallis with Dunn’s post-hoc test, when appropriate. Subjects’ clinical data were analyzed with SPSS/Win program version 17.0 (SPSS, Chicago, IL) and reported as mean ± standard deviation (SD). Differences among groups were compared using One-way ANOVA or Student’s *t*-test for continuous variables and χ^2^ test for categorical variables. Receiver Operating Characteristic (ROC) curves were calculated to estimate the area under the curve (AUC) for selected miRNAs in relation to disease (SA and UA).

Hierarchical clustering was performed based on Euclidean distance between the two CAD groups vs. controls with complete linkage method and discriminant analysis was used to calculate the percentage of cases correctly classified by combination of selected miRNAs. Probability values lower than 0.05 were considered statistically significant. The reported *p*-values were two-tailed in all calculations.

## Results

### Screening Phase

We enrolled a total of 53 consecutive CAD patients with Stable or Unstable Angina. A cohort of 20 subjects matched for sex, age, smoking habit, hypertension, and diabetes served as Control group. An initial screening of 367 miRNAs was conducted on plasma samples from 4 randomly allocated subjects from each group. The characteristics of enrolled patients and controls are summarized in Table 1. SA and UA patients significantly differed only for the number of stents implanted (0.9±1.0 vs. 1.6±0.8, respectively, p=0.019). 

**Table 1:** Clinical characteristics of enrolled subjects.

	**CONTROLS**	**STABLE**	**UNSTABLE**	
	**(n=20)**	**(n=34)**	**(n=19)**	**p**
**Gender (% Males)**	95	88.2	78.9	0.365
**Age (mean ± st.dev.)**	62.5 ± 2.1	60.0 ± 10.6	64.7 ± 11.8	0.871
**Hypertension (%)**	55.0	64.7	77.8	0.416
**Smoker (%)**	60.0	72.7	65.6	0.649
**Diabete mellitus (%)**	10.0	11.8	27.8	0.201
**History of AMI/STROKE/PCI/CABG/PTA (%)**	/	44.1	50.0	0.654
**β-blocker (%)**	/	61.8	61.1	0.892
**ASA (%)**	/	44.1	50.0	0.562
**ACE-inhibitor (%)**	/	32.4	33.3	0.708
**Diuretic (%)**	/	5.9	11.1	0.256
**Statin (%)**	/	58.8	38.9	0.214
**Nitrates (%)**	/	44.1	44.4	0.805
**Total cholesterol (mg/dL)**	/	214.2 ± 51.1	206.7 ± 51.3	0.491
**LDL cholesterol (mg/dL)**	/	148.7 ± 79.9	168.1 ± 153.3	0.678
**HDL cholesterol (mg/dL)**	/	55.3 ± 30.3	45.8 ± 12.0	0.194
**Triglycerides**	/	158.3 ± 73.1	119.8 ± 78.7	0.105
**n STENT**	/	0.9 ± 1.0	1.6 ± 0.8	0.039 *
**n vessels (stenosis > 75%)**	/	1.9 ± 1.2	1.9 ± 1.1	0.841
**CK-MB (ng/mL)**	/	4.0 ± 4.4	2.8 ± 2.9	0.267
**CK (UI/L)**	/	116.2 ± 89.6	120.7 ± 105.7	0.961
**TnI (ng/mL)**	/	0.04 ± 0.05	0.02 ± 0.01	0.122

All values are presented as mean ± standard deviation (St. dev.). Abbreviations: AMI = Acute Myocardial Infarction; PCI = percutaneous coronary intervention; CABG = coronary artery bypass graft; PTA = Percutaneous Transluminal Angioplasty; ASA = acetylsalicylic acid; CK-MB = Creatine Kinase-MB; TnI = Troponin I; * p<0.05.

Out of the 367 miRNAs screened, 178 were detectable in all subjects. By comparing the 3 cohorts of subjects, we selected 10 miRNAs which showed an average 2-fold change between CAD patients and controls. There was no evidence of differentially regulated miRNAs between SA and UA patients.

### Confirmation step and analysis of expression

**Figure 1:**
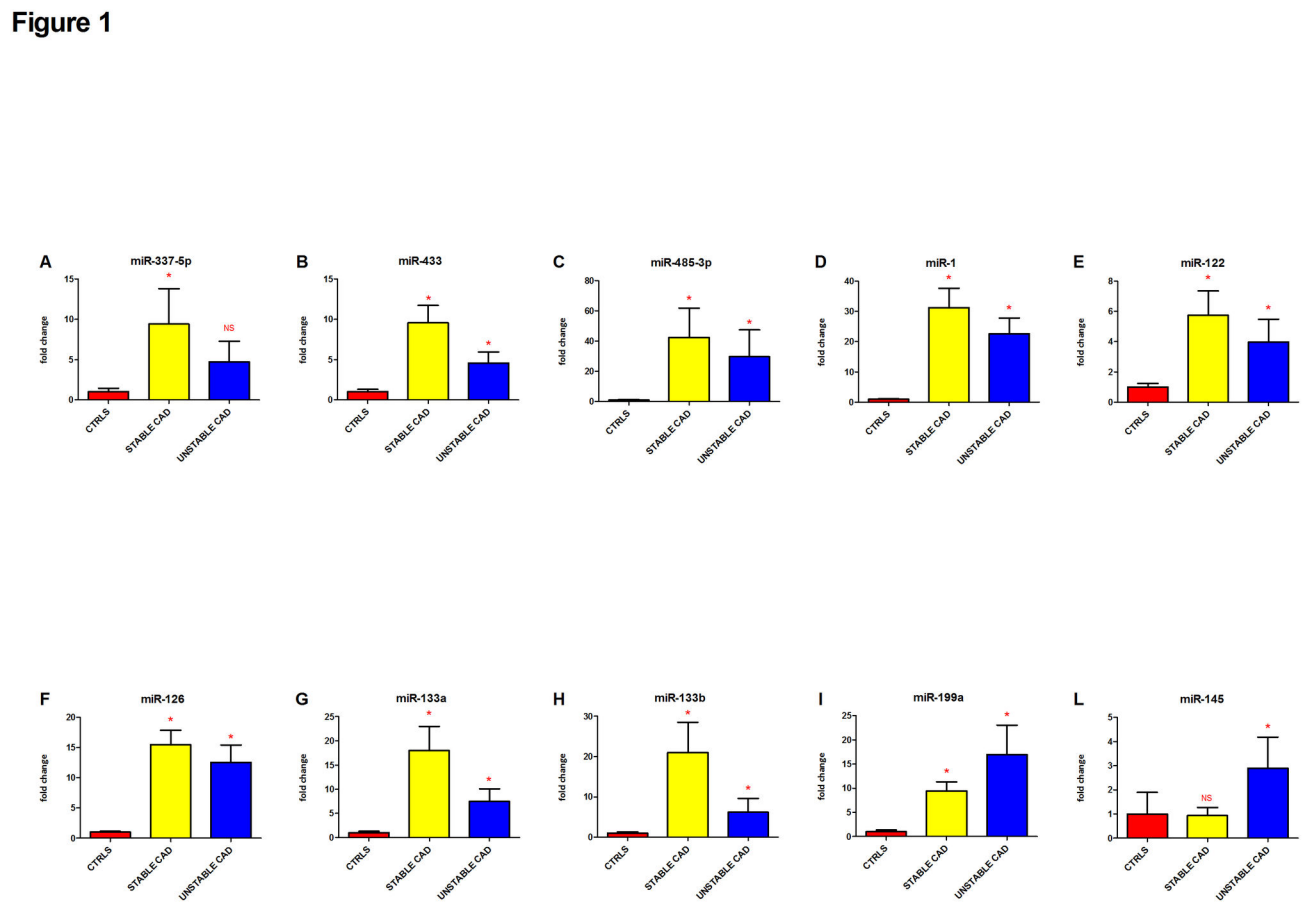
Plasmatic miRNAs regulation by CAD in Stable and Unstable Angina patients. Panels A to L. All investigated miRNAs showed increased expression in CAD subjects, both in SA (yellow bars, 5.7–42.4 fold increase with the exception of miR-145, not regulated) and UA (blue bars, 2.9–29.7 fold increase with the exception of miR-337-5p, not regulated) patients. Values indicate fold changes (expressed as mean ± SEM) of each miRNA vs. its level in control healthy subjects (CTRLS, red bars), arbitrarily set to 1. (*) indicates p≤0.05 vs. control; NS=not significant. Differences among groups were compared using either One-way ANOVA or non-parametric Kruskal-Wallis with Dunn’s post-hoc test, when appropriate.

The confirmation step of the screening phase was conducted on restricted cohorts of SA and UA patients and control subjects (n=10 for each group). Three out of the 10 putatively modulated miRNAs showed a statistically significant (p<0.05) up-regulation: miR-337-5p, -433, and -485-3p. Conversely, the other 7 miRNAs selected from the screening were not confirmed as significant at this step. The discrepancies between screening and validation results could be attributable to the differences in detection and normalization methods and in the sample sizes. The expression analysis was then extended to all remaining patients and controls and this step further uphold the significant positive regulation of miR-337-5p, -433, and -485-3p. In particular, when compared to controls, miR-337-5p (Figure 1A) showed a significant increase in the SA group (9.4±4.4 folds, p=0.001), but not in UA subjects. Conversely, miR-433 (Figure 1B) and miR-485-3p (Figure 1C) showed a very strong positive modulation both in SA (9.6±2.1 and 42.4±19.5 folds, respectively, p<0.001 for both) and in UA patients (4.6±1.3 and 29.7±17.8 folds, p=0.002 and p<0.001, respectively), when compared to controls. Interestingly, none of the 3 miRNAs showed statistically significant expression differences between SA and UA patients.

### Analysis of literature-selected miRNAs

In addition to the above screening-selected miRNAs, we investigated by qRT-PCR the expression of a subset of circulating miRNAs, previously demonstrated by us and others to be involved in CAD [14] and STEMI [8]. In particular, we analyzed the expression of CAD-related miR-17-5p, -92a, -126, -133a, -145, -155, -199a, and -208a, and STEMI-related miR-1, -122, -133a, -133b, -375, and -499-5p. Six of these miRNAs showed an increase in both SA and in UA patients (Figure 1D to 1L): miR-1 (31.2±6.5 in SA, 22.6±5.2 in UA, p<0.001 for both; Figure 1D), miR-122 (5.7±1.6 in SA, 4.0±1.5 in UA, p=0.040 and p=0.011, respectively; Figure 1E), miR-126 (15.4±2.4 in SA, 12.5±2.9 in UA, p<0.001 for both; Figure 1F), miR-133a (18.0±5.0 in SA, 7.4±2.6 in UA, p<0.001 for both; Figure 1G), miR-133b (21.0±7.5 in SA, 6.2±3.4 in UA, p=0.003 and p<0.001, respectively; Figure 1H), and miR-199a (9.4±1.9 in SA, 16.9±6.1 in UA, p=0.001 and p=0.007, respectively; Figure 1I). In contrast, miR-145 demonstrated a positive regulation in UA patients only (2.9±1.3, p=0.002; Figure 1L). 

### Diagnostic potential of circulating miRNAs

**Figure 2:**
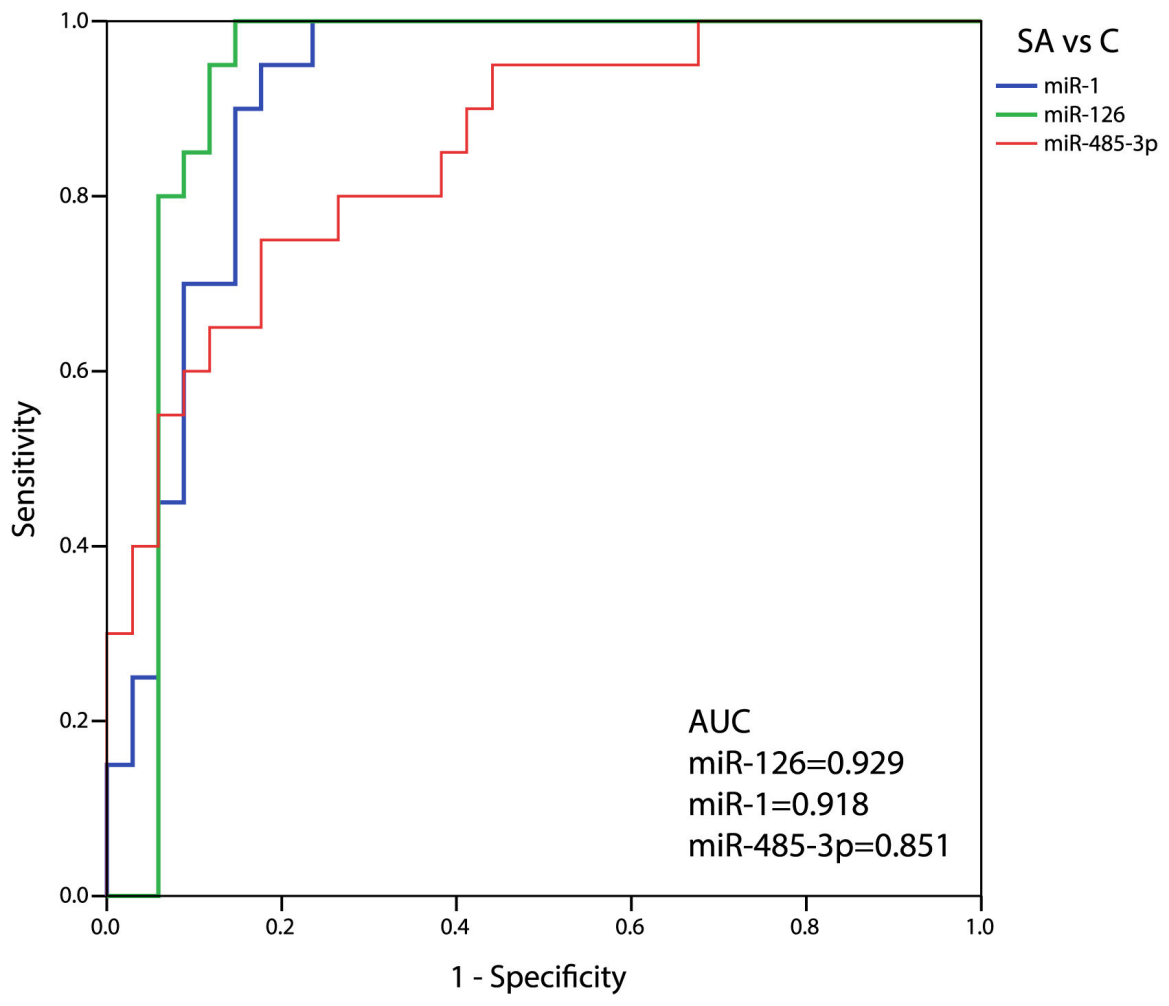
ROC curve analysis of CAD-miRNAs in Stable Angina patients and control subjects. The figure depicts calculated ROC curve and respective AUC values for miR-1, miR-126, and miR-485-3p, which exhibited good accuracy (AUC>0.85) in differentiating Stable Angina (SA) patients from matched controls (C).

**Figure 3:**
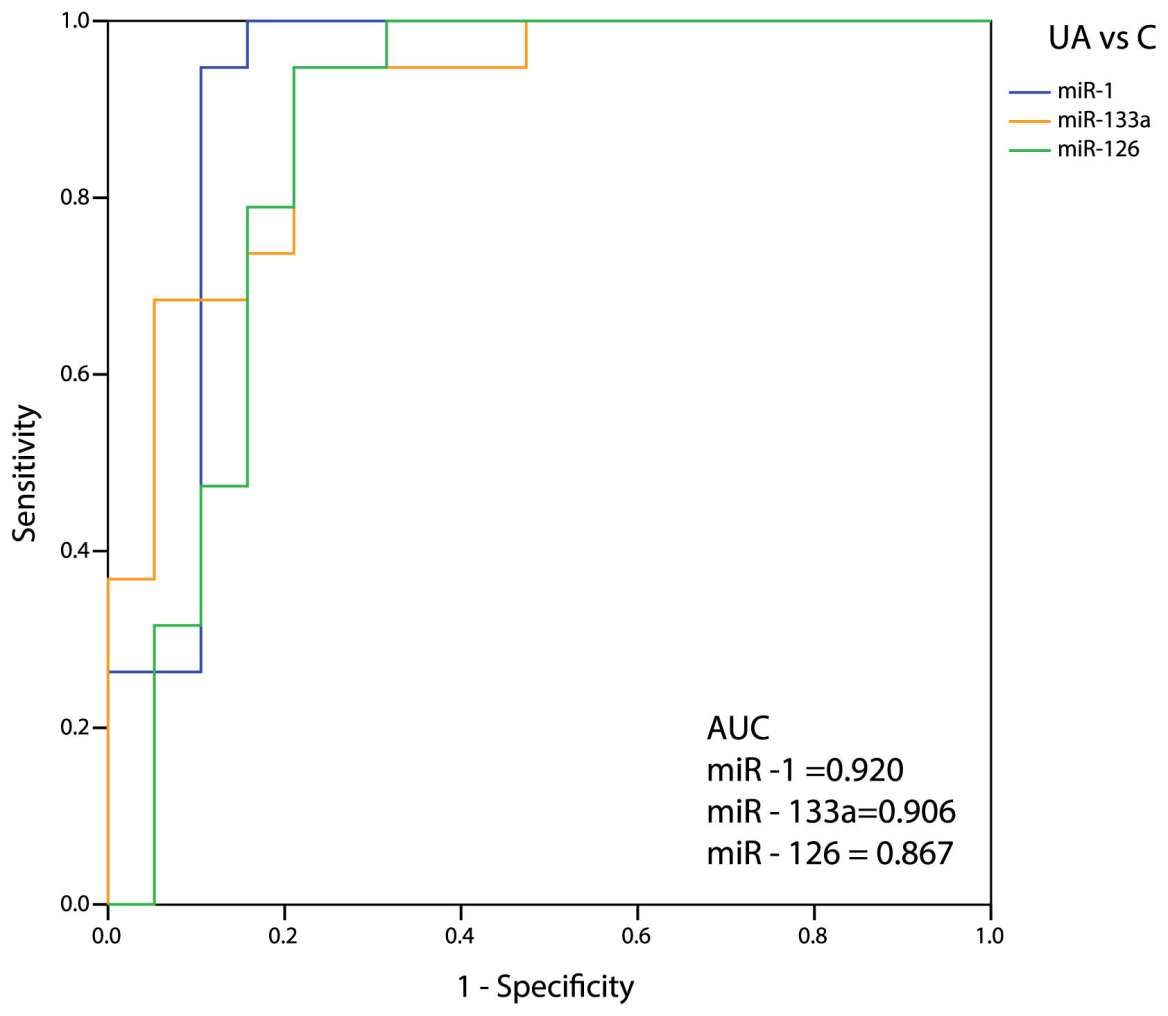
ROC curve analysis of CAD-miRNAs in Unstable Angina patients and control subjects. The figure depicts calculated ROC curve and respective AUC values for miR-1, miR-126, and miR-133a, which exhibited good accuracy (AUC>0.85) in differentiating Unstable Angina (UA) patients from matched controls (C).

**Figure 4:**
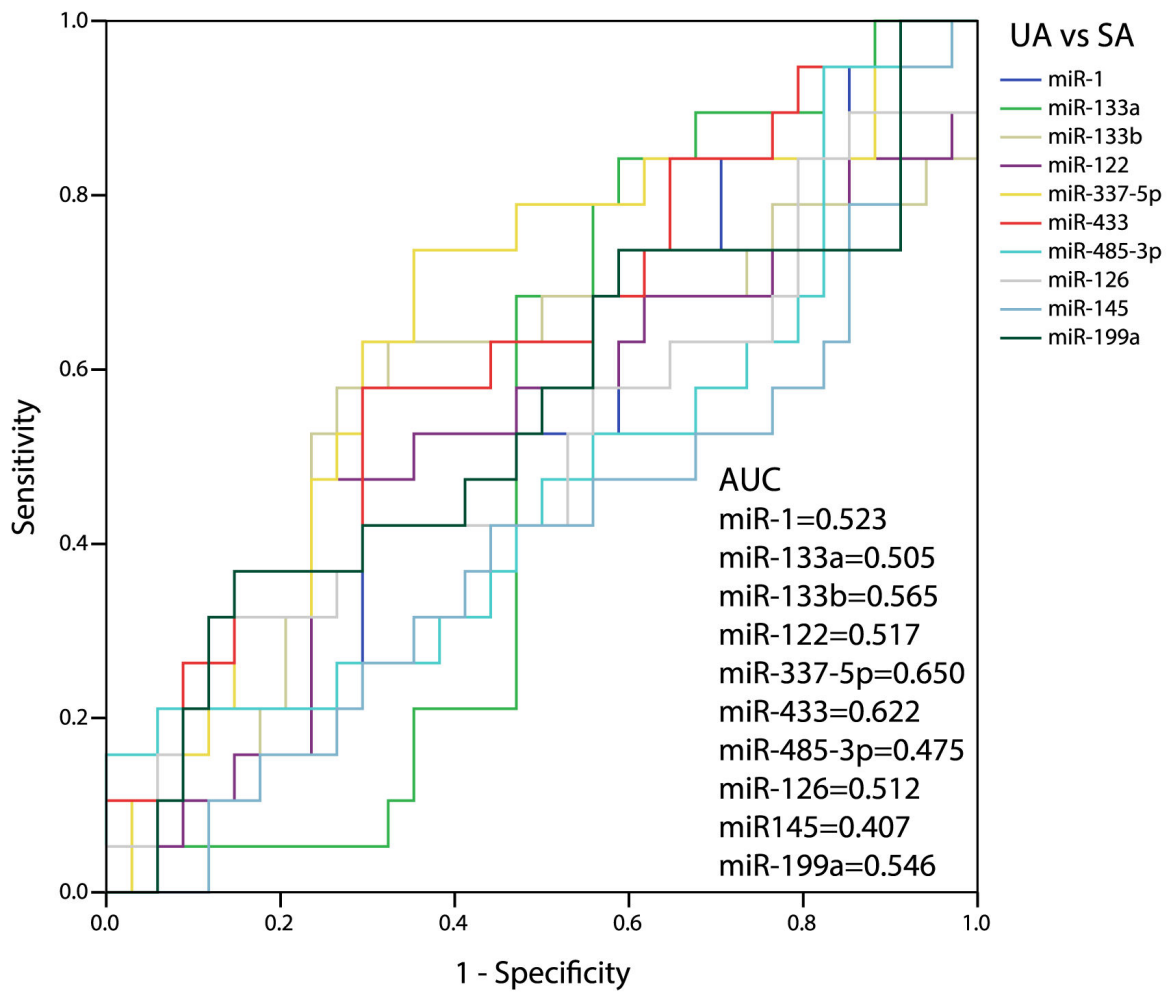
ROC curve analysis of CAD-miRNAs in Stable and Unstable Angina patients. None of the investigated miRNAs exhibited adequate accuracy in differentiating Stable (SA) from Unstable Angina (UA) patients: AUC values ranged between 0.404 (miR-145) and 0.678 (miR-337-5p).

To determine the predictive capacity of miRNAs identified above, we performed ROC curve analyses. The most predictive AUC values (>0.85) were obtained in SA patients, when compared to Controls, for miR-1, -126, and -485-3p (0.918, 0.929, and 0.851, respectively; Figure 2), although good AUC values were obtained for almost all considered miRNAs (Figure S1). Similarly, AUC values >0.85 were observed in UA subjects for miR-1, -126, and -133a (0.920, 0.867, and 0.906, respectively; Figure 3), while all remaining miRNAs showed AUC values <0.85 (Figure S2). Interestingly, none of the considered miRNAs reached an acceptable AUC value to differentiate SA from UA patients (Figure 4), ranging from 0.404 (miR-145) to 0.678 (miR-337-5p).

**Figure 5:**
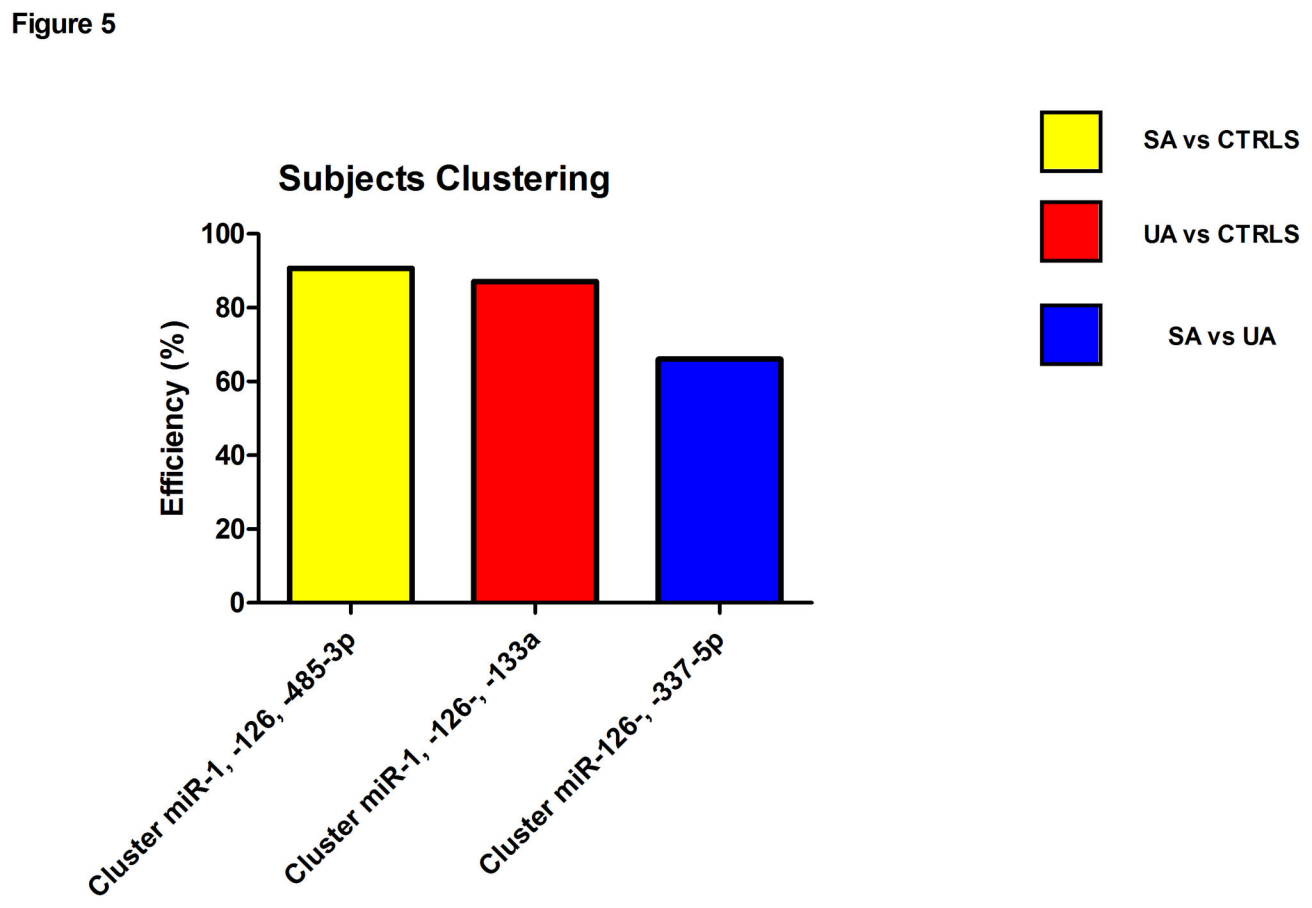
miRNA clusters efficiently differentiate SA and UA patients from Controls. Hierarchical clustering demonstrated that different miRNA “signatures” efficiently classify between matched controls (CTRLS) and CAD patients. The cluster composed by miR-1 and miR-126 and miR-485-3p can be used to correctly classify SA patients from controls with 90.2% (yellow bar) efficiency. Similarly the miR-1, miR-126 and miR-133a cluster can be used to correctly classify UA patients from controls with 87.2% (red bar) efficiency. No signatures of miRNAs could be found to efficiently discriminate SA from UA patients with an accuracy > 66% (miR-126 and miR-337-5p cluster, blue bar).

In an attempt to identify a signature with diagnostic potential resulting from a combination of miRNAs, we performed a hierarchical cluster analysis on all miRNAs found regulated by CAD. Figure 5 (and Figure S3) shows that the combined expression of miR-1, -126, and -485-3p correctly classifies SA patients vs. Controls with an efficiency of 90.2%. Similarly, the combination of miR-1, -133a, and -126 presented 87.2% efficiency in discriminating UA patients from control subjects (Figure 5 and Figure S4). In contrast, the efficiency of CAD-regulated circulating miRNAs to correctly differentiate SA from UA subjects was not higher than 66% (see Figure 5). 

## Discussion

The main findings of this work are the evidences that: i) specific plasmatic miRNA signatures may have the potential to unveil with high efficiency (>85%) the presence of a significant angiographically documented Stable (miR-1, miR-126, miR-485-3p) or Unstable (miR-1, miR-126, miR133a) CAD vs. matched Controls; ii) conversely, the ability of plasmatic miRNAs to discriminate Stable from Unstable CAD is low. 

MiRNAs have recently gained interest for their role in the pathogenesis of cardiovascular disease, including atherosclerosis and acute coronary syndromes [15]. In the search for diagnostic and predictive biomarkers of CAD, circulating miRNAs have been previously investigated in blood, serum, plasma, platelets, and PBMC. A large number of miRNAs have been found to be either up- or down-regulated in patients with angiographically-documented significant Stable and Unstable CAD compared with controls. However, differences in study design, patient population, and, particularly, miRNA sources, are likely to be responsible for the high variability and poor overlap of the aberrant miRNA signatures identified, thus making difficult to extrapolate conclusive information. In particular, since miRNA profiles are likely to be specific for the investigated blood components [10], the starting material is crucial for the evaluation of the potential of miRNAs as circulating biomarkers of CAD. In view of a possible clinical transferability, we have isolated circulating miRNAs from EDTA-plasma, given the sensitivity of the TaqMan qPCR technique in these preparations [14,16]. Further, plasma allows the exclusion of the strong interference of PMBC and platelets as sources of RNA, given the high heterogeneity of miRNAs signatures previously identified in these components [10]. To our knowledge, a previous study by Fichtlscherer and coworkers [14] is the only one that investigated the plasmatic levels of miRNAs from patients with CAD. Thirty-six patients with stable angiographically-documented CAD and 7 healthy controls served as derivation cohort; results obtained were then prospectively tested in 14 negative controls and 31 patients. Circulating levels of endothelial-enriched miR-126, miR-17, and miR-92a, inflammation-associated miR-155 and muscle-enriched miR-145 have been found significantly reduced in CAD compared with controls. In contrast, cardiac-muscle-enriched miR-133a and miR-208a showed a tendency for an increased concentration in plasma, albeit not significant. Notably, there are some important differences between Fichtlscherer’s study and ours, which should be considered when comparing the results. First, along with stable CAD patients, we have prospectively enrolled a cohort of troponin-negative angiographically-documented unstable patients. Secondly, we have used a different normalization methodology that relies on an internal normalizer instead of a spiked-in synthetic exogenous miRNA (e.g. cel-miR-39). In our opinion, our technique allows a more reliable identification of a suitable and stable internal normalizer which compensates for the variability of RNA amount in different samples. The alternative spike-in method, more suited for normalization of differences in RNA recovery, has intrinsic flaws, because it postulates that equal volumes of plasma always contain the same amount of RNA. Thus, the addition of a fixed amount of an exogenous synthetic miRNA to variable amounts of total RNA may affect the final miRNA quantification [17]. Finally and most importantly, we put attention during the enrollment phase to match stable and unstable patients for known cardiovascular risk factors and pharmacological therapy, given the influence of these variables on circulating miRNAs [18-21].

The discriminating power of selected and validated CAD-related miRNAs was firstly assessed by means of ROC curve analysis. We considered as highly discriminating AUC values those above 0.85 [22]. MiR-1, miR-126, and miR-133a were found to be highly predictive as potential single biomarker of both stable and unstable angina. Interestingly, miR-1 and miR-133a are co-transcribed and have been previously extensively reported as associated to cardiovascular disease [23]. Intriguingly, the plasmatic up-regulation of the “endothelial-specific” miR-126 in presence of stable and unstable angina may mirror a condition of endothelial dysfunction occurring in the ischemic microcirculation during chronic angina or acute coronary syndromes [24]. There is no known relation between miR-485-3p expression and cardiovascular disease.

It is noteworthy mentioning that, using our own normalization technique, Sun and co-workers [25] recently reported the decrease in plasmatic expression of miR-126 in CAD patients with high low-density lipoprotein (LDL) cholesterol levels. In contrast, the level of miR-126 was significantly increased when LDL cholesterol was high in patients who had risk factors for CAD but did not have angiographically significant CAD. Different from our study and in analogy with Fichtlscherer’s study, however, patient enrolment was performed in a population unmatched for age and major cardiovascular risk factors, and patients without significant coronary stenosis served as internal control group. These dissimilarities in patient’s selection may at least partly explain the discrepancy in results with our study. 

 MiRNA signatures in tissues and plasma have been shown in oncologic patients to be able to efficiently predict disease development and aggressiveness [26]. We have tested by means of a hierarchical cluster analysis the hypothesis that plasma miRNA signatures have the potential to unveil CAD. The combination of cardiac-enriched miR-1, endothelial-enriched miR-126, and miR-485-3p allowed the best discrimination between SA patients and control donors with good efficiency. The addition to this cluster of the cardiac-enriched miR-133a with exclusion of miR-485-3p identified successfully the presence of UA. These results may have a relevant clinical read-out, given the unmet need of circulating diagnostic biomarkers particularly in patients with stable angina [27]. 

 We failed to identify any circulating miRNA (either by ROC curve or cluster analysis) with a satisfying potential in discriminating SA from UA. The two signatures overlapped for miR-1 and miR-126, and only differed for the presence of miR-485-3p and miR-133a in SA and UA patients, respectively. We and others have previously described an aberrant circulating miRNAs regulation in presence of acute coronary syndromes with Troponin dispersion [28]. Although it has been suggested that unstable angina may entail a higher expression of miRNA levels [29], SA and UA patients did not show a markedly diverse circulating miRNA profile in our clinical setting. It is worth to note that our UA population was carefully documented for its troponin-negative profile. 

A relevant issue is whether plasmatic signatures elicited in angina are specific of troponin-negative CAD and not features in common with MI. Interestingly, a data lookup from our previous results in STEMI [8] and NSTEMI [9] showed that: i) miR-1 and miR-133a were found increased both in angina and MI patients; ii) miR-126 and miR-485-3p were up-regulated in patients with angina only; iii) miR-499-5p was observed specifically raised in MI patients only. A potential drawback of the aforementioned comparison lies in the use of different internal standards (miR-17-5p for MI and miR-16 for CAD groups). Nonetheless, a retrospective analysis of published MI plasma signatures showed that a restricted cohort of miRNAs have been repeatedly found dysregulated (namely miR-1, miR-133a, and miR-499-5p), irrespectively of the methodology of normalization adopted (see Table S1), and this is in full agreement with our observations. Notably, the aberrant plasmatic expression of miR-499-5p emerges as the most consistent marker of occurrence of heart damage [8,9,30,31]. Although the exact mechanism by which miRNAs are released into the bloodstream upon myocardial ischemia is a matter of investigation [32], this feature may be explained with a passive release of this specific miRNA triggered by cardiomyocyte death. Thus, mir-499-5p represents a potential discriminating factor within plasma miRNA signatures in CAD between Troponin-positive vs. negative coronary syndromes [23]. Confirmatory studies are warranted in the future to verify this issue under standardized experimental settings. 

The main limitation of this work is its single-center set-up involving a limited number of patients. Multicenter large-scale studies will be required to definitively assess the potential of circulating miRNAs as diagnostic biomarkers of angina. Further, our screening method allowed the detection of only 367 human miRNAs: we cannot exclude that other miRNAs not present on our arrays may be modulated under the same experimental conditions. This work should, thus, be regarded of a proof-of-principle that specific circulating miRNAs may discriminate CAD patients from matched controls and, as such, have the potential to be used as reliable diagnostic biomarkers.

 In conclusion, this work shows that plasmatic profiles of miRNAs are capable to accurately identify the presence of troponin-negative angiographically-documented CAD, causing either SA or UA. Due to the high overlap of the signatures pinpointed, the miRNAs discriminating power between SA and UA was found to be low. Further studies should address with larger population cohorts the potential utility of plasmatic miRNAs as biomarkers of CAD, along with the mechanisms underpinning their release in the bloodstream. 

## Supporting Information

Figure S1 **ROC curve analysis of CAD-miRNAs in Stable Angina patients and control subjects presenting AUC values <0.85.** The figure depicts calculated ROC curve and respective AUC values for miR-122, miR-133a, miR-133b, miR-145, miR-199a, miR-337-5p, and miR-433, which exhibited acceptable accuracy (0.72<AUC<0.85) in differentiating Stable Angina (SA) patients from matched controls (C).
(JPG)

Figure S2 **ROC curve analysis of CAD-miRNAs in Unstable Angina patients and control subjects presenting AUC values <0.85.** The figure depicts calculated ROC curve and respective AUC values for miR-122, miR-133b, miR-145, miR-199a, miR-337-5p, miR-433 and miR-485-3p, which exhibited acceptable accuracy (0.69<AUC<0.85) in differentiating Unstable Angina (UA) patients from matched controls (C).
(JPG)

Figure S3 **Hierarchical clustering of stable angina patients and healthy controls using microRNA signatures.** The figure depicts a dendrogram representing unsupervised classification of SA patients and control subjects basing on combination of miR-1, miR-126, and miR-485-3p expression. SA=Stable Angina, C=Controls.
(JPG)

Figure S4 **Hierarchical clustering of unstable angina patients and healthy controls using microRNA signatures.** The figure depicts a dendrogram representing unsupervised classification of SA patients and Control subjects basing on combination of miR-1, miR-126, and miR-133a expression. UA=Unstable Angina, C=Controls.
(JPG)

Table S1 **Analysis of previous works concerning circulating miRNA regulation after Myocardial Infarction.**
(DOCX)
