# Supplementary figures and images for: Diagnostic Potential of Plasmatic MicroRNA Signatures in Stable and Unstable Angina
Source: PLoS One. 2013 Nov 15;8(11):e80345. doi: 10.1371/journal.pone.0080345 (PMC3829878; doi:10.1371/journal.pone.0080345)

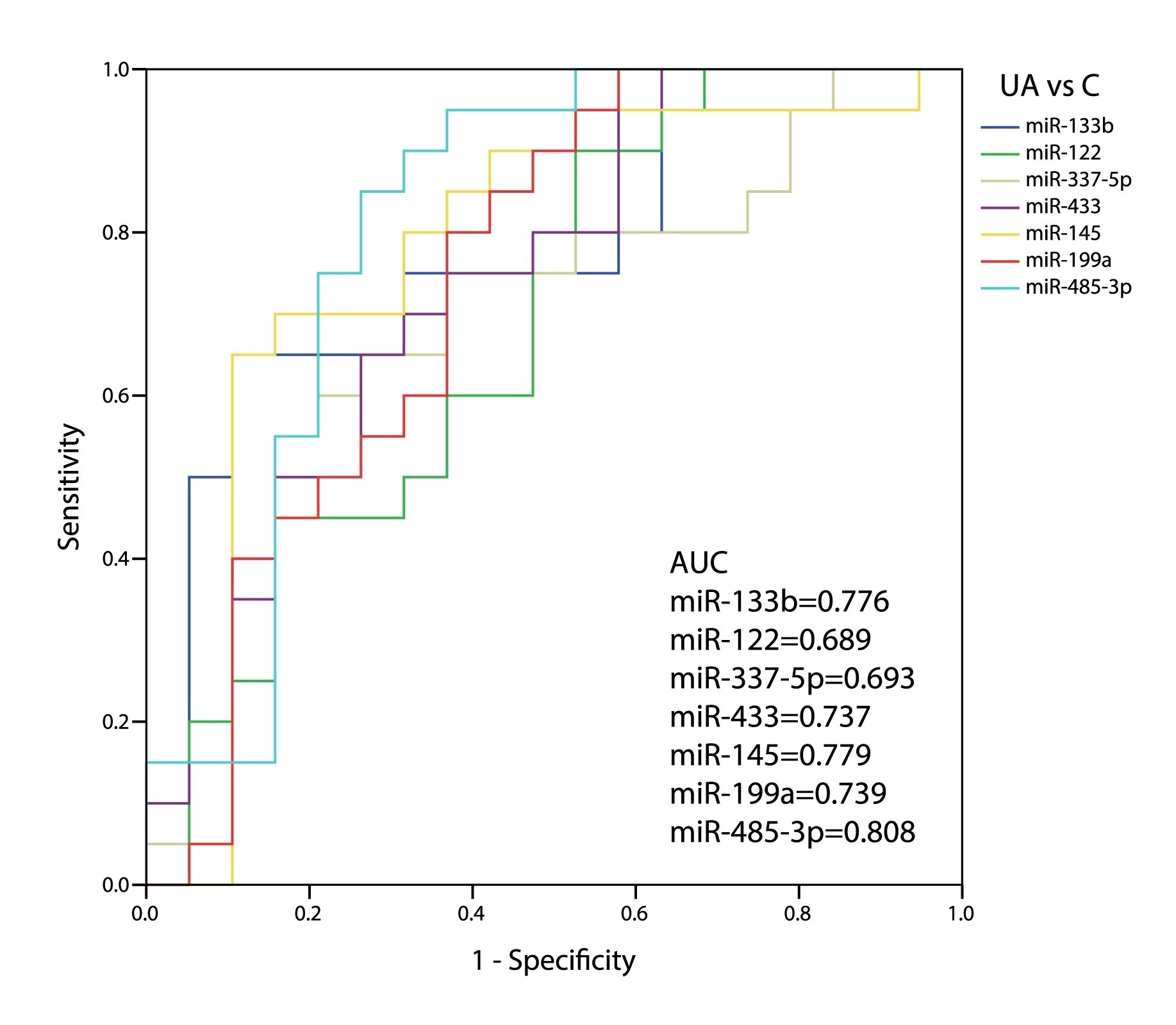

Supplement: Figure S1 — ROC curve analysis of CAD-miRNAs in Stable Angina patients and control subjects presenting AUC values <0.85. The figure depicts calculated ROC curve and respective AUC values for miR-122, miR-133a, miR-133b, miR-145, miR-199a, miR-337-5p, and miR-433, which exhibited acceptable accuracy (0.72<AUC<0.85) in differentiating Stable Angina (SA) patients from matched controls (C). (JPG) [file pone.0080345.s001.jpg]

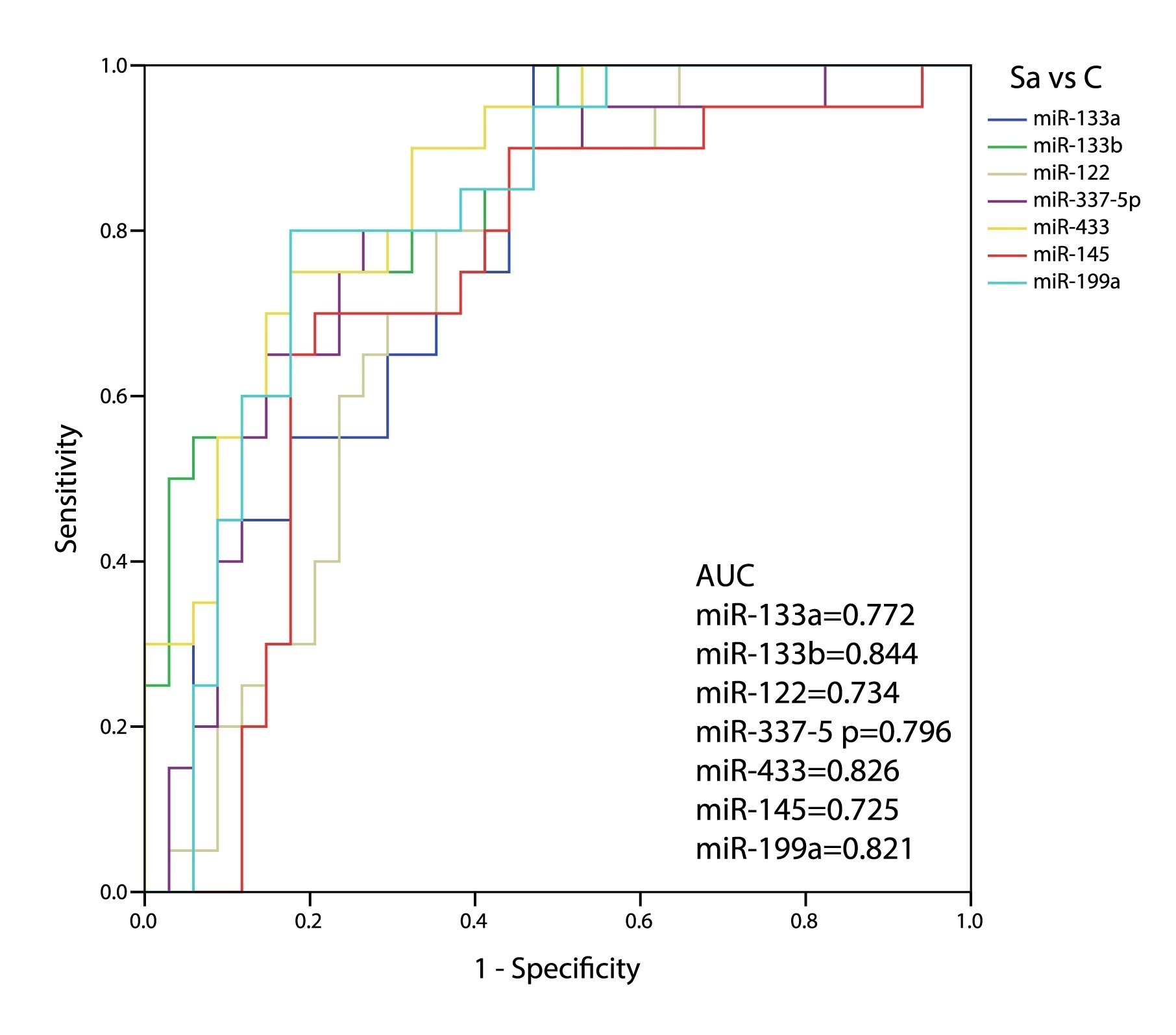

Supplement: Figure S2 — ROC curve analysis of CAD-miRNAs in Unstable Angina patients and control subjects presenting AUC values <0.85. The figure depicts calculated ROC curve and respective AUC values for miR-122, miR-133b, miR-145, miR-199a, miR-337-5p, miR-433 and miR-485-3p, which exhibited acceptable accuracy (0.69<AUC<0.85) in differentiating Unstable Angina (UA) patients from matched controls (C). (JPG) [file pone.0080345.s002.jpg]

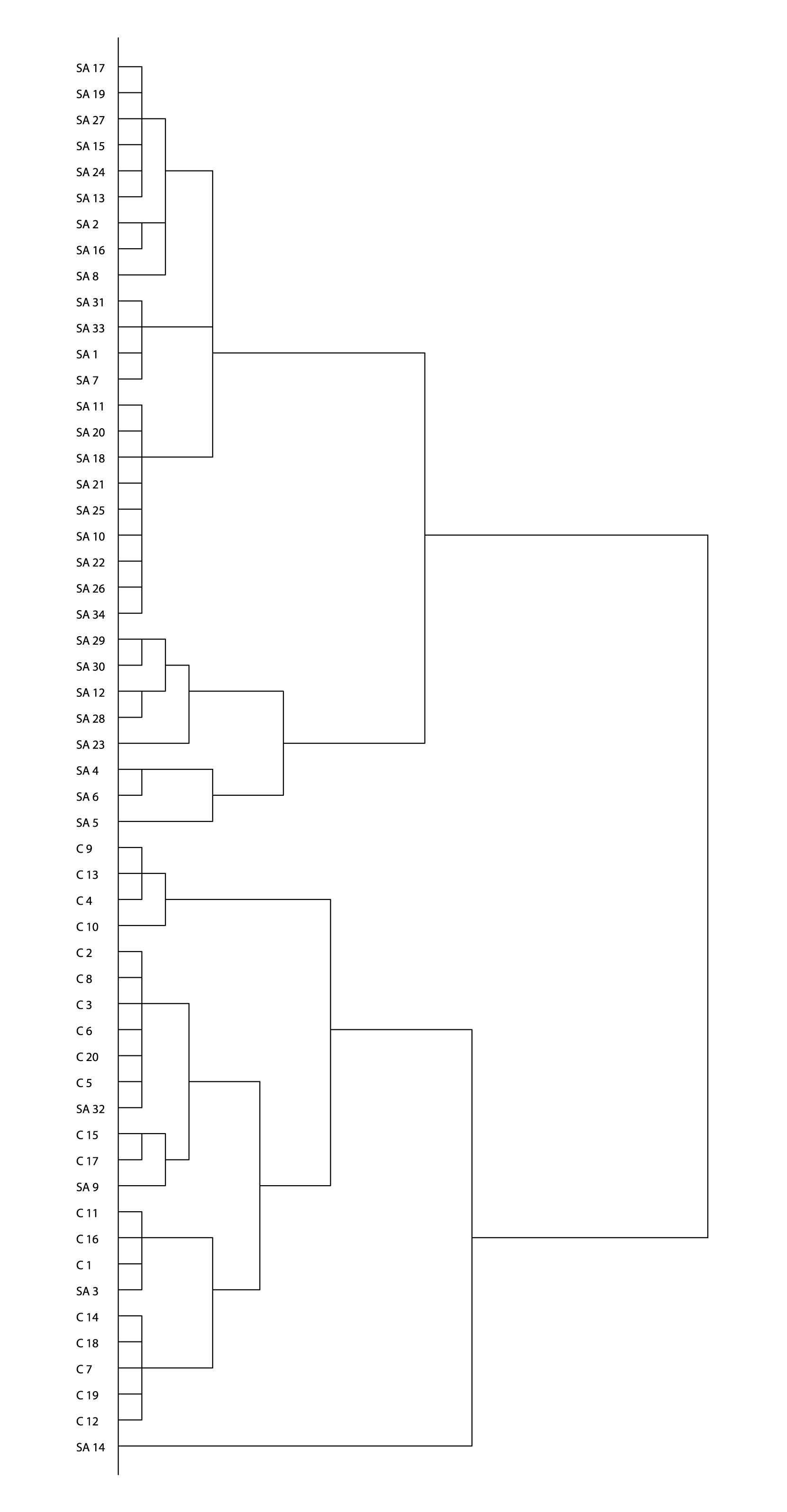

Supplement: Figure S3 — Hierarchical clustering of stable angina patients and healthy controls using microRNA signatures. The figure depicts a dendrogram representing unsupervised classification of SA patients and control subjects basing on combination of miR-1, miR-126, and miR-485-3p expression. SA=Stable Angina, C=Controls. (JPG) [file pone.0080345.s003.jpg]

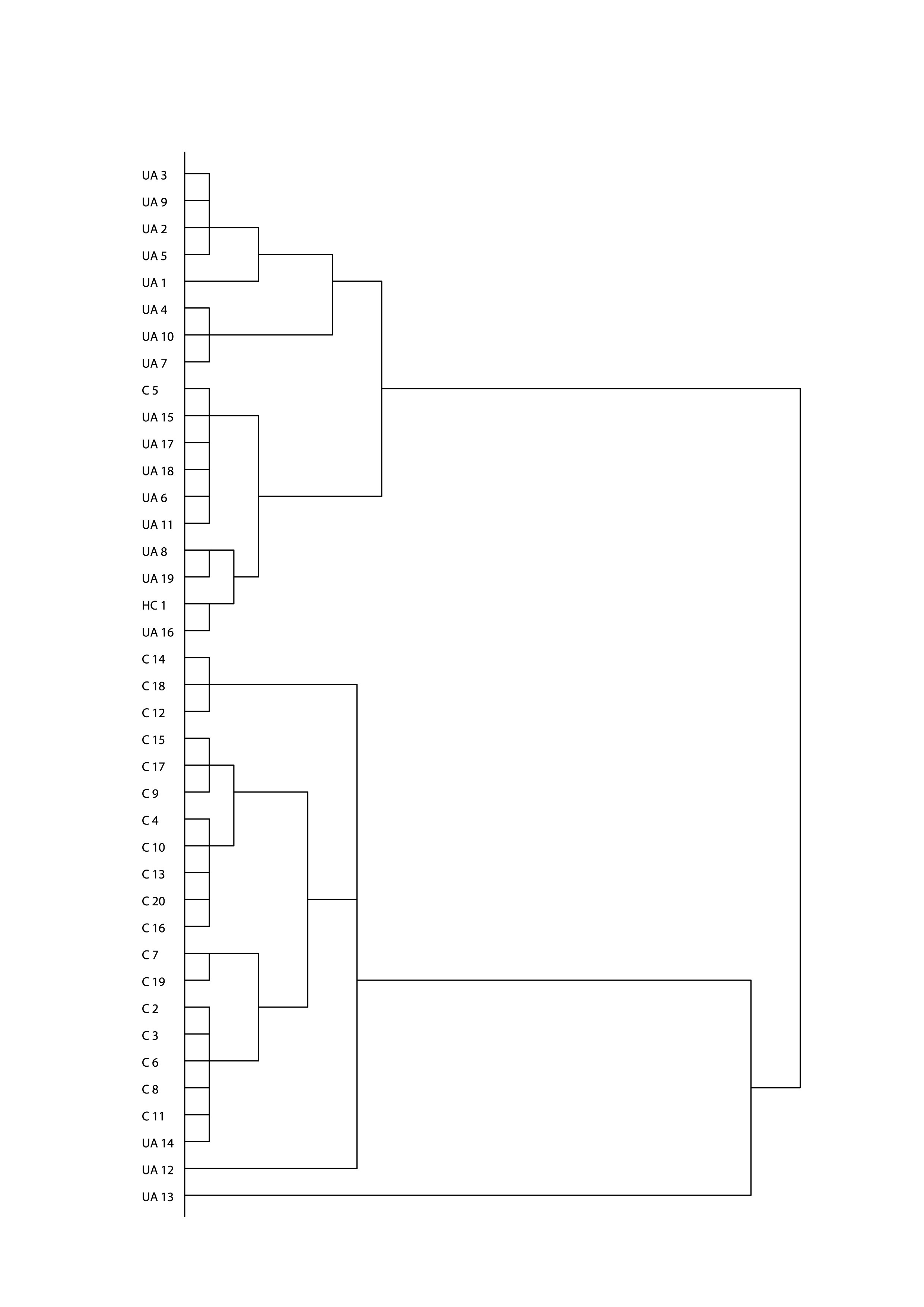

Supplement: Figure S4 — Hierarchical clustering of unstable angina patients and healthy controls using microRNA signatures. The figure depicts a dendrogram representing unsupervised classification of SA patients and Control subjects basing on combination of miR-1, miR-126, and miR-133a expression. UA=Unstable Angina, C=Controls. (JPG) [file pone.0080345.s004.jpg]
