# Supplementary material for: Diagnostic Potential of Plasmatic MicroRNA Signatures in Stable and Unstable Angina
Source: PLoS One. 2013 Nov 15;8(11):e80345. doi: 10.1371/journal.pone.0080345 (PMC3829878; doi:10.1371/journal.pone.0080345)
Supplement: Table S1 — Analysis of previous works concerning circulating miRNA regulation after Myocardial Infarction. (DOCX) [file pone.0080345.s005.docx]

| MI-Regulated miRNAs | Source | Normalization | Reference |
| --- | --- | --- | --- |
| miR-1, miR-208b and miR-499-5p | Plasma | miR-17 | [1] |
| miR-1, miR -21, miR -133a, miR-423-5p and miR-499-5p | Plasma | miR-17-5p | [2] |
| miR-208b and miR-499-5p | Plasma | Synthetic miRNA spike-in | [3] |
| miR-499, miR-133a and miR-208a | Plasma | Synthetic miRNA spike-in | [4] |
| miR-1, miR-133a, miR-208b and miR-499-5p | Plasma | No normalization | [5] |
| miR-1, miR-133a, miR-208b, miR-223 and miR-499-5p | Plasma | Synthetic miRNA spike-in | [6] |
| miR-1, miR-133a, miR-133b, and miR-499-5p | Plasma | miR-17-5p | [7] |
| miR-499-5p | Plasma | Synthetic miRNA spike-in | [8] |
| miR-1, miR-133a, miR-208a and miR-499 | Plasma | Synthetic miRNA spike-in | [9] |

**Supplementary Table S1.** Analysis of previous works concerning circulating miRNA regulation after Myocardial Infarction

MI: Myocardial Infarction

**Supplementary References**

1) Gidlöf O, Smith JG, Miyazu K, Gilje P, Spencer A, et al. (2013) Circulating cardio-enriched microRNAs are associated with long-term prognosis following myocardial infarction. BMC Cardiovasc Disord 28;13:12.

2) Olivieri F, Antonicelli R, Lorenzi M, D'Alessandra Y, Lazzarini R, et al. (2013) Diagnostic potential of circulating miR-499-5p in elderly patients with acute non ST-elevation myocardial infarction. Int J Cardiol 167:531-6.

3) Devaux Y, Vausort M, Goretti E, Nazarov PV, Azuaje F, et al. (2012) Use of circulating microRNAs to diagnose acute myocardial infarction. Clin Chem 58:559-67.

4) De Rosa S, Fichtlscherer S, Lehmann R, Assmus B, Dimmeler S, et al. (2011) Transcoronary concentration gradients of circulating microRNAs. Circulation 24:1936-44

5) Gidlöf O, Andersson P, van der Pals J, Götberg M, Erlinge D () Cardiospecific microRNA plasma levels correlate with troponin and cardiac function in patients with ST elevation Myocardial infarction, are selectively dependent on renal elimination, and can be detected in urine samples. Cardiology 118:217-26

6) Corsten MF, Dennert R, Jochems S, Kuznetsova T, Devaux Y et al. (2010) Circulating MicroRNA-208b and MicroRNA-499 reflect myocardial damage in cardiovascular disease. Circ Cardiovasc Genet 3:499-506.

7) D'Alessandra Y, Devanna P, Limana F, Straino S, Di Carlo A, et al. (2010) Circulating microRNAs are new and sensitive biomarkers of myocardial infarction. Eur Heart J 31:2765-73.

8) Adachi T, Nakanishi M, Otsuka Y, Nishimura K, Hirokawa G, et al. (2010) Plasma microRNA 499 as a biomarker of acute myocardial infarction. Clin Chem 56:1183-5.

9) Wang GK, Zhu JQ, Zhang JT, Li Q, Li Y, et al. (2010) Circulating microRNA: a novel potential biomarker for early diagnosis of acute myocardial infarction in humans. Eur Heart J 31:659-66.
